# Vital Signs as Predictors of Oxygen Requirements in COVID-19 Outpatients: The Development of a Streamlined Risk Score

**DOI:** 10.7759/cureus.61270

**Published:** 2024-05-28

**Authors:** Narumichi Iwamura, Kanako Tsutsumi

**Affiliations:** 1 Department of Internal Medicine, Sasebo Chuo Hospital, Sasebo, JPN

**Keywords:** respiratory rate, spo2, body temperature, omicron variant, healthcare triage, vital signs, clinical risk score, oxygen therapy, sars-cov-2, covid-19

## Abstract

Background: With COVID-19 becoming a common disease, primary care facilities such as clinics are required to efficiently triage patients at high risk of severe illness within the constraints of limited medical resources. However, existing COVID-19 severity risk scores require detailed medical history assessments, such as evaluating the severity of pneumonia via chest CT and accounting for past and comorbid conditions. Therefore, they may not be suitable for practical use in clinical settings with limited medical resources, including personnel and equipment.

Purpose: The goal is to identify key variables that predict the need for oxygen therapy in COVID-19 patients and develop a simplified clinical risk score based solely on vital signs to predict oxygen requirements.

Patients and methods: A retrospective observational study of 584 outpatients with COVID-19 confirmed by polymerase chain reaction test visited Sasebo Chuo Hospital between April 28, 2022, and August 18, 2022. Analyses were conducted after adjustment for background factors of age and sex with propensity score matching. We used the Fisher test for nominal variables and the Kruskal-Wallis test for continuous variables.

Results: After adjusting for age and sex, several factors significantly correlated with the need for oxygen within seven days including body temperature (p < 0.001), respiratory rate (p = 0.007), SpO_2_ (p < 0.001), and the detection of pneumonia on CT scans (p = 0.032). The area under the receiver-operating characteristic curve for the risk score based on these vital signs and CT was 0.947 (95% confidence interval: 0.911-0.982). The risk score based solely on vital signs was 0.937 (0.900-0.974), demonstrating the ability to predict oxygen administration with no significant differences.

Conclusions: Body temperature, advanced age, increased respiratory rate, decreased SpO_2_, and the presence of pneumonia on CT scans were significant predictors of oxygen need within seven days among the study participants. The risk score, based solely on vital signs, effectively and simply assesses the likelihood of requiring oxygen therapy within seven days with high accuracy. The risk score, which utilizes only age and vital signs and does not require a detailed patient history or CT scans, could streamline hospital referral processes for admissions.

## Introduction

Severe acute respiratory syndrome coronavirus 2 (SARS-CoV-2) was identified as a novel coronavirus in December 2019 in Wuhan, Hubei Province, China. It is the causative agent of coronavirus disease 2019 (COVID-19), a novel viral infection [1]. As the number of patients with COVID-19 increases, primary care clinics and hospitals will play an even greater role in efficiently triaging those individuals. These facilities are tasked with selecting patients who currently or in the future require oxygen administration and making decisions about referring them to a hospital for admission. Predicting the deterioration of patients with COVID-19 in advance is crucial in medical care, and while numerous studies have been conducted on this topic, there is relatively little research in Japan. Various risk factors associated with the severity of SARS-CoV-2 have been reported in the COVIREG-JP registry (n = 3376; January 16, 2020, to May 31, 2020). The factors (odds ratios by multivariate analysis) associated with a higher proportion requiring oxygen administration on admission were chronic lung disease (2.51), male sex (2.09), obesity (1.75), cardiovascular disease (1.48), diabetes (1.34), and hypertension (1.33) [2]. As the strain targeted in that study was a Delta strain inferred from the observation period, it is unclear whether the results of that study can be applied to the SARS-CoV-2 Omicron strains. With the increasing number of patients with COVID-19, efforts to develop early prognostic scores for predicting the severe form of the disease can assist in allocating limited healthcare resources. A prognostic score, as described in the COVID-19 Practice Guide [3], to predict the requirement of oxygen administration based on the analysis of COVIREGI-JP involves assessing age, sex, and the presence or absence of up to 13 evaluation items: age, sex, body mass index (BMI), congestive heart failure, cerebrovascular disease, diabetes, hypertension, malignancy, fever, cough, dyspnea, wheezing, and fatigue. This scoring system is designed for inpatient departments, which differs from the current forms of medical treatment for COVID-19; most patients are treated in an outpatient setting. Because these prognostic scores were developed based on the study of SARS-CoV-2 in Wuhan and Delta strains during the early stages of the COVID-19 pandemic, it remains uncertain whether these scores can be applied to SARS-CoV-2 Omicron strains. Thus, we considered that criteria are needed to accurately and efficiently determine the need for referral to an inpatient hospital in COVID-19 outpatient care. Initially, we identified risk factors associated with oxygen administration required within seven days among patients with SARS-CoV-2 Omicron strains after adjusting for background factors of age and sex through propensity score matching. Based on these results, we endeavored to establish a clinical risk score for predicting the requirement of oxygen administration within seven days.

This article was previously posted to the Research Square preprint server on March 15, 2023.

## Materials and methods

Purpose

To identify factors predicting the requirement of oxygen administration within seven days in patients with SARS-CoV-2 Omicron strains and establish a clinical risk score for predicting the required oxygen administration within seven days.

Target patients

**Figure 1:**
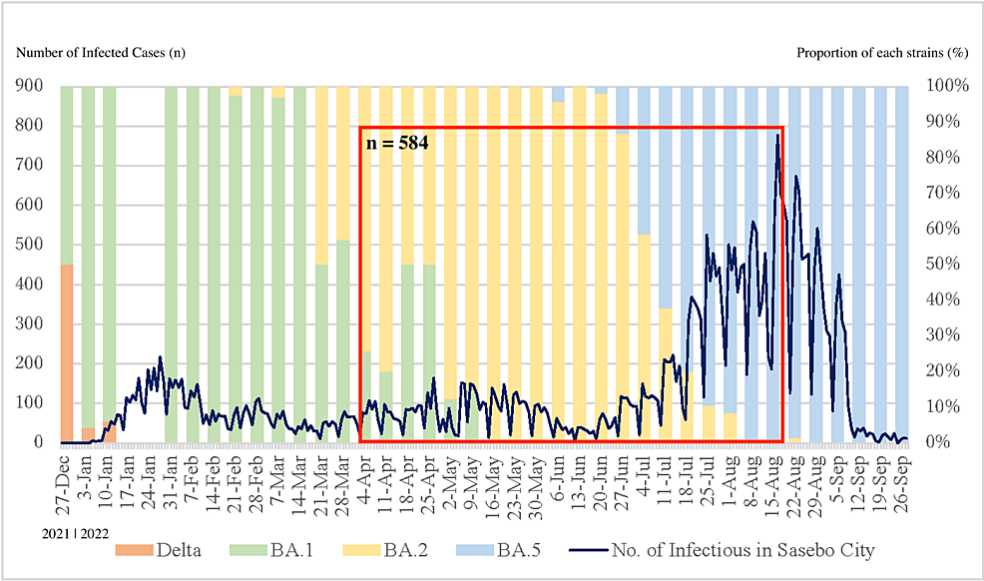
Number of new infections in Sasebo city and results of genome analysis in Nagasaki

This is a retrospective observational study. We included data from 584 patients with COVID-19 confirmed by polymerase chain reaction (PCR) tests who visited Sasebo Chuo Hospital in Nagasaki Prefecture, Japan, between April 4, 2022, and August 18, 2022 (Figure 1).

The dominant strains of SARS-CoV-2 during this period were estimated as BA.2 and BA.5 based on genome analysis research conducted by the Prefectural Environmental Health Research Center in this area. The inclusion criteria were as follows: (1) patients with COVID-19 complaining of breathlessness, severe malaise, or fever of >39℃ lasting >3 days; (2) patients aged ≥65 years; (3) patients aged between 40 and 64 years with risk factors for severe illness (unvaccinated or vaccinated only once, history of COPD, diabetes, hyperlipidemia, hypertension, chronic kidney disease, malignant cancer, smoking, obesity with a BMI of >30 kg/m², or immunodeficiency after solid organ transplantation); (4) children with underlying medical conditions. We excluded patients who were already undergoing oxygen therapy at the time of inclusion from this study. The number of patients who did not require oxygen within seven days (n = 562) and those requiring oxygen within seven days (n = 22) was determined. After adjusting for background factors of age and sex through propensity score matching, patients who did not require oxygen within seven days (n = 38) and those requiring oxygen within seven days (n = 19) were analyzed. The patients’ characteristics are summarized in Table 1.

**Table 1:** Baseline demographic and disease characteristics * Race and ethnic group were determined from the patients' names. † Any risk factors for COVID-19 were reported by the patients. †† Duration of symptoms was reported by the patients. § Severity classification was based on Japanese guidelines on novel coronavirus as follows [4]: mild, SpO_2 _≧ 96% and no pneumonia findings; moderateⅠ, 93%<SpO_2_<96% or pneumonia findings; moderate Ⅱ, SpO_2 _≦ 93% or requiring oxygen administration.

Characteristics		Before matching of age and sex (n = 584)	After matching of age and sex (n = 57)
Presumptive strain of SARS-CoV-2 - n (%)			
	BA.2	355 (61)	20 (35)
	BA.5	229 (39)	37 (65)
Age			
	Median (range) - yr	54 (39-69)	79 (73-87)
	≤15 yr - n (%)	38 (6.5)	0 (0.0)
	≥65 yr - n (%)	187 (32)	53 (93)
	>70 yr - n (%)	133 (23)	46 (81)
Male sex - n (%)		262 (45)	32 (56)
Race or ethnic group - no./total n (%)*			
	White	2 (0.34)	0 (0.0)
	Black	0 (0.0)	0 (0.0)
	Asian	581 (99)	57 (100)
	Mixed race	1 (0.17)	0 (0.0)
BMI - median (range)		22.5 (20.1-25.4)	21.5 (19.7-23.9)
Any risk factor for COVID-19 progression - n (%)^†^			
	Age ≥ 55 yr	282 (48)	57 (100)
	Diabetes	71 (12)	11 (19)
	Obesity: BMI > 30	38 (6.5)	1 (1.8)
	Chronic kidney disease	19 (3.2)	5 (8.8)
	Congestive heart failure	12 (2.1)	3 (5.3)
	Chronic obstructive pulmonary disease	6 (1.0)	2 (3.5)
	Asthma	61 (10)	5 (8.8)
No. of concurrent risk factors for COVID-19 progression - n (%)			
	0	223 (38)	0 (0.0)
	1	252 (43)	34 (60)
	2	91 (16)	19 (33)
	3	17 (2.9)	4 (7.0)
	4	1 (0.17)	0 (0.0)
No. of times vaccinated - n (%)			
	0	128 (22)	8 (14)
	1	8 (1.4)	0 (0.0)
	2	139 (24)	5 (8.8)
	3	296 (51)	40 (70)
	4	18 (3.1)	4 (7.0)
Duration of symptoms - n (%)^††^			
	≤3 days	460 (79)	43 (75)
	≥4 days	124 (21)	14 (25)
No. of days hospitalized			
	0 days - n (%)	499 (85)	30 (53)
	1-11 days - n (%)	62 (11)	17 (30)
	≥12 days - n (%)	23 (3.9)	10 (18)
Severity classification^§^			
	minor - n (%)	361 (62)	20 (35)
	moderate-Ⅰ - n (%)	205 (35)	24 (42)
	moderate-Ⅱ - n (%)	18 (3.1)	13 (23)
	severe - n (%)	0 (0.0)	0 (0.0)
	death - n (%)	0 (0.0)	0 (0.0)
No. of confirmation of pneumonia on CT - n (%)		224 (38)	37 (65)
No. of oxygen administration required - n (%)		22 (3.8)	19 (33)

Methods

The primary endpoint was set as the requirement of oxygen administration within seven days. We adopted SpO_2_ below 93% as one of the criteria for requiring oxygen administration. However, in some cases, we did not administer oxygen to patients whose SpO_2_ was clearly below 93% even under normal conditions due to underlying diseases such as chronic obstructive pulmonary disease (COPD). Meanwhile, we administered oxygen to patients who complained of respiratory distress although their SpO_2_ was above 93% and to patients whose CT pneumonia was severe and who were expected to have a lower SpO_2 _in the future. Outpatients deemed able to return home were instructed to measure their SpO_2_ frequently at home with a pulse oximeter. Patients without a pulse oximeter were provided one by the health care center. They were asked to return to the hospital if their SpO_2_ dropped below 93% on the pulse oximeter or if they noticed symptoms such as respiratory distress. Oxygen was administered by nasal cannula if <4 L/min and by a reservoir mask if between 5 and 10 L/min. Nasal high flow was used if SpO_2 _could not be maintained above 93% even after administering oxygen at ≥ 10 L/min. We set the outcome as not hospitalization but oxygen administration to exclude patients hospitalized not because of the aggravation of COVID-19. Body temperature was measured in the axilla using a medical thermometer. The association of the primary endpoint and the following 25 variables was statistically analyzed: BMI of >30 kg/m², history of COVID-19 infection, age of ≥65 years, history of immunosuppressive drug administration, asthma and smoking, administration or under follow-up of anticancer drugs, hypertension, dyslipidemia, severity classification in Japan, congestive heart failure, sex, diabetes, confirmation of pneumonia on CT, chronic kidney disease, COPD, BMI, body temperature, number of concurrent risk factors for COVID-19 progression [5], respiratory rate, number of times vaccinated, number of days stayed in the hospital, age, and duration of symptoms. Analyses were conducted after adjustment for background factors of age and sex with propensity score matching. We used the Fisher test for nominal variables and the Kruskal-Wallis test for continuous variables.

Risk scores were calculated based on data in the chart at the time of visiting the outpatient clinic. The scores, referencing odds ratios from logistic analysis, were derived using body temperature, age, respiratory rate, SpO_2_, and confirmation of pneumonia on CT, which were relevant in the analysis of method 1. The test characteristics of this risk score were analyzed. Scores with and without confirmation of pneumonia on CT as evaluation items were compared based on the area under the receiver-operating characteristic curve (AUC). We derived a risk score according to prior literature [6,7]. EZR (Saitama Medical Center Jichii Medical University, Saitama, Japan) was used for all statistical analyses in this study [8].

## Results

Before adjusting for background factors of age and sex, age ≥ 65 years (p < 0.001), severity classification (p < 0.001), pneumonia images on CT (p < 0.001), chronic kidney disease (p = 0.004), body temperature (p < 0.001), number of risk factors for COVID-19 progression (p < 0.001), respiratory rate (p < 0.001), SpO_2_ (p < 0.001), number of times vaccinated (p = 0.027), number of days hospitalized (p < 0.001), age (p < 0.001), duration of symptoms (p = 0.004) were found to be significantly associated with oxygen administration required within seven days. After adjusting for background factors of age and sex, severity classification (p < 0.001), diabetes (p < 0.003), confirmation of pneumonia on CT (p < 0.032), body temperature (p < 0.001), respiratory rate (p < 0.007), SpO_2_ (p < 0.001), hospitalization (p < 0.001), duration of symptoms (p = 0.017) were found to be significantly associated with oxygen administration required within seven days (Table 2).

**Table 2:** Examination of variables associated with oxygen administration required within seven days * Fisher test, † Kruskal–Wallis test, and a p-value of less than 0.05 is considered significant.

Factor	Group	Oxygen Administration Required		P-value
		No (n = 38)	Yes (n = 19)	
BMI > 30*	No	38 (100)	18 (94.7)	0.333
	Yes	0 (0.0)	1 (5.3)	
History of COVID-19 infection*	No	38 (100)	19 (100)	NA
	Yes	0 (0.0)	0 (0.0)	
Age ≥ 65 yr*	No	3 (7.9)	0 (0.0)	0.544
	Yes	35 (92)	19 (100)	
Immunosuppressive drugs administration*	No	34 (89)	18 (95)	0.655
	Yes	4 (11)	1 (5.3)	
History of asthma*	No	37 (97)	16 (84)	0.103
	Yes	1 (2.6)	3 (16)	
History of smoking*	No	24 (63)	11 (58)	0.777
	Yes	14 (37)	8 (42)	
Anticancer drug administration or cancer under follow-up*	No	31 (82)	18 (95)	0.247
	Yes	7 (18)	1(5.3)	
Hypertension*	No	11 (29)	10 (53)	0.092
	Yes	27 (71)	9 (47)	
Dyslipidemia*	No	26 (68)	12 (63)	0.769
	Yes	12 (32)	7 (37)	
Severity classification*	minor	15 (40)	2 (11)	<0.001
	moderate Ⅰ	23 (61)	4 (21)	
	moderate Ⅱ	0 (0.0)	13 (68)	
Congestive heart disease*	No	36 (95)	18 (95)	1
	Yes	2 (5.3)	1 (5.3)	
Male*	No	17 (45)	9 (48)	1
	Yes	21 (55)	10 (53)	
Diabetes*	No	25 (66)	19 (100.0)	0.003
	Yes	13 (34)	0 (0.0)	
Confirmation of pneumonia on CT*	No	15 (40)	2 (11)	0.032
	Yes	23 (61)	17 (89)	
Chronic kidney cancer*	No	35 (92.1)	17 (89)	1
	Yes	3 (7.9)	2 (11)	
Chronic obstructive pulmonary disease*	No	37 (97)	18 (95)	1
	Yes	1 (2.6)	1 (5.3)	
BMI^†^		21.5 [19.7, 23.8]	21.7 [19.9, 23.9]	0.986
Body temperature^†^		36.7 [36.5, 36.9]	37.7 [37.3, 38.5]	<0.001
No. of concurrent risk factors for COVID-19 progression^†^		1.00 [1.00, 2.00]	1.00 [1.00, 2.00]	0.554
Respiratory rate^†^		20.0 [18.0, 20.0]	20.0 [20.0, 22.0]	0.007
SpO_2_^†^		97.0 [96.0, 98.0]	94.0 [90.5, 96.0]	<0.001
No. of times vaccinated^†^		3.00 [3.00, 3.00]	3.00 [2.50, 3.00]	0.726
No. of days hospitalization^†^		0.00 [0.00, 0.00]	11.0 [7.00, 17.5]	<0.001
Age^†^		77.0 [73.0, 87.0]	78.0 [73.5, 86.5]	0.806
Duration of symptoms^†^		2.00 [1.00, 4.00]	1.00 [0.00, 2.00]	0.017

Following the methods of previous studies [6,7], we established a risk score for oxygen administration required within seven days based on body temperature (<37.5℃; 0 points, 37.5℃-38.4℃; 4 points, ≥38.5℃; 7 points), age (<70; 0 points, ≥70; 5 points), respiratory rate (<20/min; 0 points, ≥20/min; 2 points), and SpO_2_ (≥96%; 0 points, <96%; 1 point) (Table 3).

**Table 3:** Selected predictor variables for multivariable model of predicting oxygen administration required within seven days * Fisher test, † Chai-squared test, and a p-value of less than 0.05 is considered significant. AUC: area under the receiver-operating characteristic curve

Evaluation Items of Clinical Risk Score		No. of Oxygen Administration Required/Total No. of Patients (%)	P-value	Odds Ratio (95% CI)	AUC (95% CI)	No. of Risk Points for Oxygen Administration Required
Body temperature	≤37.4℃	2/443 (0.451)	<0.001*	1	0.864 (0.795-0.934)	0
	37.5℃-38.4℃	10/92 (10.9)		26.9 (5.79-125)		4
	≥38.5℃	10/49 (20.4)		56.5 (12-267)		7
Age	≤69 yr	2/441 (0.454)	<0.001^†^	1	0.845 (0.782-0.909)	0
	≥70 yr	20/143 (14.0)		35.8 (8.25-155)		5
Respiratory rate	≤19/min	2/352 (0.568)	<0.001^†^	1	0.766 (0.701-0.831)	0
	≥20/min	20/232 (8.62)		16.5 (3.82-71.3)		2
SpO_2_	≥96%	11/535 (2.06)	<0.001*	1	0.716 (0.609-0.824)	0
	≤95%	11/49 (22.4)		13.8 (5.62-33.9)		1
Pneumonia images on CT	No	2/360 (0.556)	<0.001^†^	1	0.773 (0.708-0.838)	0
	Yes	20/224 (8.93)		17.5 (4.06-75.8)		2

**Figure 2:**
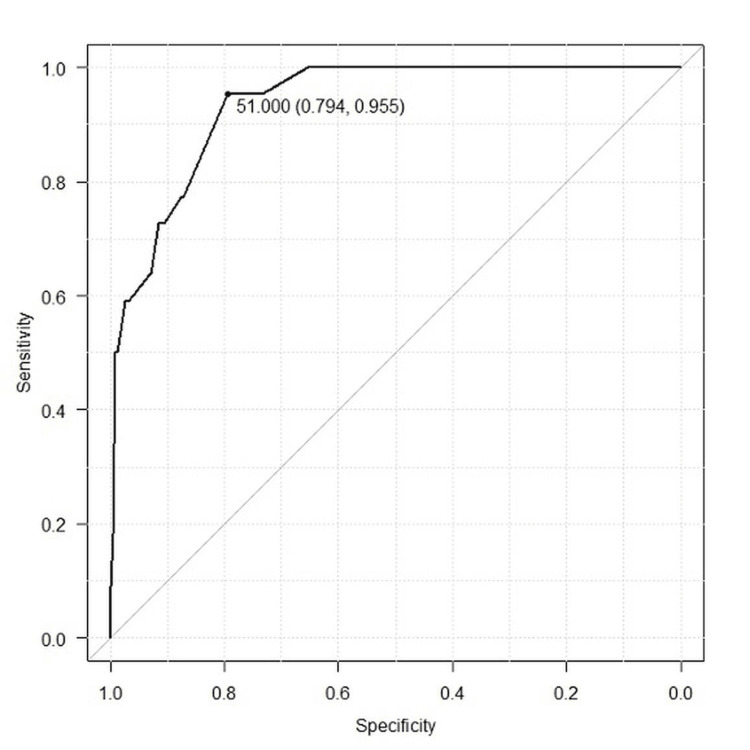
ROC curve of a risk score for predicting oxygen administration required within seven days based on the following evaluation items: body temperature, age, respiratory rate, and SpO2. ROC curve: receiver-operating characteristic curve

This risk score could screen for oxygen administration required within seven days with a sensitivity/specificity of 100%/65% and a positive/negative likelihood ratio of 2.9/0.0 when the cutoff was set at 5 points. It was able to diagnose the requirement of oxygen administration within seven days with a sensitivity/specificity of 59%/97% and a positive/negative likelihood ratio of 18/0.40 when the cutoff was set at 10 points. The AUC for this risk score in the study population was 0.937 (95% confidence interval (CI): 0.900-0.974) (Figure 2).

**Figure 3:**
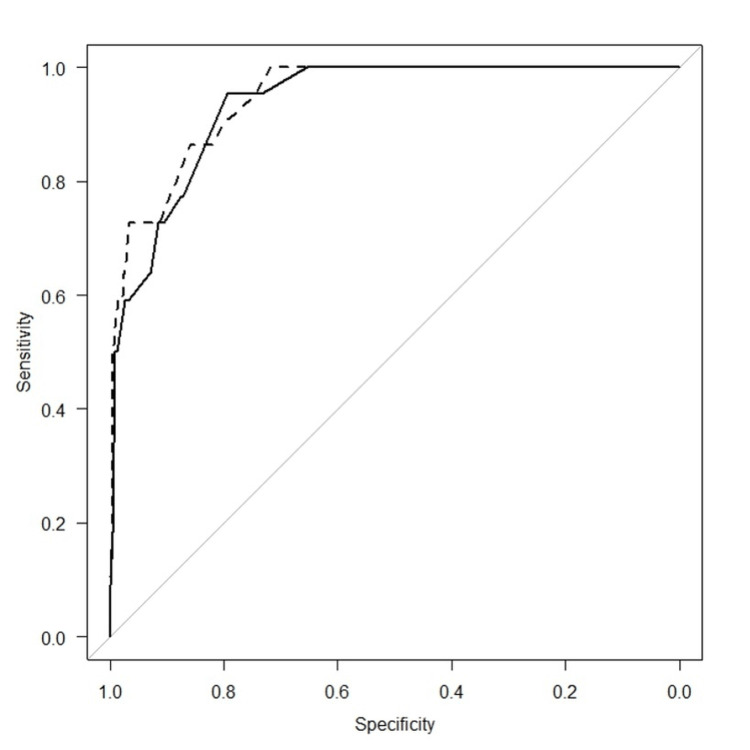
Comparison of two ROC curves of a risk score for predicting oxygen administration required within seven days on the following evaluation items: body temperature, age, respiratory rate, and SpO2 (A) vs. body temperature, age, respiratory rate, SpO2, and pneumonia images on CT (B). ― The risk score without confirmation of pneumonia on CT. --- The risk score with confirmation of pneumonia on CT. ROC curve: receiver-operating characteristic curve

Meanwhile, the AUC of the risk score, which included body temperature (4 points for a temperature between 37.5°C and 38.4°C and 7 points for a temperature above 38.5°C), age (5 points for age over 70 years), respiratory rate (2 points for ≥20 breaths), SpO_2_ (1 point for SpO_2_ below 95%), and confirmation of pneumonia on CT (2 points for pneumonia with a CT image), was 0.947 (95% CI: 0.911-0.982). No significant difference was detected between the AUC of the risk score with confirmation of pneumonia on CT and that of the risk score without confirmation of pneumonia on CT (p = 0.28) (Figure 3).

## Discussion

With the increasing number of patients with COVID-19, the focus of COVID-19 care has shifted from inpatient wards to outpatient settings. Clinics and medical hospitals, especially those providing primary health care, play an increasingly crucial role in the proper and efficient triage of patients with COVID-19. These facilities carefully select patients with a current or potential requirement for oxygen administration and make decisions about referring them to a hospital for admission. In this context, there is an increasing need for a risk score to triage patients with COVID-19 in an outpatient setting. However, most existing risk scores assume serious illness in inpatients, and they often comprise several components for practical use in an outpatient setting. Furthermore, most COVID-19 severity risk scores require detailed medical history interviews, laboratory tests, and sometimes chest CT scans to assess the severity of pneumonia. As a result, they are not suitable for triage in clinics or emergency rooms with limited medical resources [9-11]. The present study aimed to address these challenges.

In the target group before adjusting for background factors (562 vs. 22), age > 65 years, confirmation of pneumonia on CT, chronic kidney disease, body temperature, number of risk factors for COVID-19 progression, respiratory rate, SpO_2_, number of times vaccinated, age, and duration of symptoms were associated with oxygen administration required within seven days. However, the potential confounding of background factors of age and sex was a concern. In the group adjusted for age and sex with propensity score matching (38 vs. 19), severity classification, diabetes, confirmation of pneumonia on CT, body temperature, respiratory rate, SpO_2_, hospitalization, and duration of symptoms were significantly associated with oxygen administration required within seven days. After age- and sex-matching, the median SpO_2_ at presentation in the group that did not require oxygen administration (n = 38) was 97.0 [96.0-98.0], while the median SpO_2_ in the group that required oxygen administration (n = 19) was 94.0 [90.5-96.0]. As mentioned in the Methods section, our institution adopted a SpO_2_ of ≤ 93% as one of the criteria for administering oxygen. Nevertheless, the median SpO_2_ of 94% at presentation in the group that required oxygen administration could be attributed to the fact that the SpO_2_ in the group that required oxygen administration declined several days after admission.

As the severity classification in Japan includes the requirement for oxygen administration as one of the criteria (Table 1), it is not surprising that severity classification was significantly associated with oxygen administration required within seven days. Abnormal vital signs, such as high temperature, increased respiratory rate, and low SpO_2_, were more likely to be associated with oxygen administration required within seven days. To our knowledge, no reports have examined the association between these vital signs and oxygen administration in patients with COVID-19, especially Omicron strains. Meanwhile, for example, high body temperature (>38°C) and increased respiratory rate (RR > 20) are included in the criteria for systemic inflammatory response syndrome. The primary cause of respiratory failure in COVID-19 is considered to be an exaggerated host immune response. Given these facts, the results relating hyperthermia and increased respiratory rate to oxygen administration required within seven days seem reasonable. It is also reasonable that patients with pneumonia on chest CT require oxygen administration within seven days. The duration of symptoms was significantly shorter in the group requiring oxygen administration within seven days, possibly due to earlier hospital presentation in more severe cases requiring oxygen within seven days. The rate of oxygen administration within seven days was significantly higher in the nondiabetic group than in the diabetic group, 43.2% (19/44) vs. 0.0% (0/13), a result that is inconsistent with clinical findings. The exact cause is unknown, but the target population may have been biased due to an inadequate sample size, requiring further study with a sufficient sample size. BMI, administration of immunosuppressive drugs, history of bronchial asthma and smoking, hypertension, dyslipidemia, heart failure, chronic kidney disease, COPD, and the number of times vaccinated were not significantly associated with oxygen administration within seven days. Concerning these results, the number of patients included in the study was insufficient to detect significant associations. Whether these factors are associated with COVID-19 severity remains controversial and may differ between SARS-CoV-2 strains, requiring further investigation with an increased sample size for each strain.

The AUC of the risk score for predicting oxygen administration required, using the evaluation items of temperature, age, respiratory rate, and SpO_2_, was 0.937 (95% CI: 0.900-0.974), indicating high accuracy in predicting oxygen administration within seven days. The AUC of the risk score, incorporating temperature, age, respiratory rate, SpO_2_, and confirmation of pneumonia on CT, was 0.947 (95% CI: 0.911-0.982), demonstrating good accuracy in predicting oxygen administration within seven days (Figure 3). Because there was no significant difference between the AUC of both risk scores, we propose a simplified risk score based on temperature, age, respiratory rate, and SpO_2_. This allows for a simpler risk assessment without the need for a CT scan or a detailed medical history interview. Although it is ideal to assess pneumonia by CT scan when determining the need for hospitalization, many medical institutions face challenges in accessing CT. Scoring based on temperature, age, respiratory rate, and SpO_2_, without confirmation of pneumonia on CT, showed comparable accuracy to scoring with confirmation of pneumonia on CT as an evaluation item. The risk score can accurately predict oxygen administration within seven days using only age and vital signs.

Nyman M et al. proposed a risk score for COVID-19-positive outpatients to predict the 30-day risk of hospitalization based on a retrospective observational study of 67,470 patients with COVID-19 confirmed by PCR test between March 12, 2020, and February 8, 2021. The Mayo Clinic COVID-19 risk score, consisting of 13 components including age, sex, chronic lung disease, congenital heart disease, congestive heart failure, coronary artery disease, diabetes mellitus, end-stage liver disease, hypertension, immunocompromised status, nursing home residence, and pregnancy, showed an AUC of 0.837 (95% CI: 0.830-0.843) for predicting admission [12]. In our study, the AUC of the risk score for predicting oxygen administration required, using the evaluation items of temperature, age, respiratory rate, and SpO_2_, was calculated as 0.937 (95% CI: 0.900-0.974). This indicates that our risk score can more efficiently and accurately predict oxygen administration required in an outpatient setting, although the outcomes are slightly different.

A risk score of <5 points could help exclude patients requiring oxygen administration within seven days with a sensitivity/specificity of 100%/65% and a positive/negative likelihood ratio of 2.9/0.0. The proportion of patients in the study who required oxygen within seven days was 3.77% (22/584). With the prior probability of 3.77%, the positive/negative predictive value with a cutoff of 5 points was calculated as 10.1%/100%. Meanwhile, a risk score of ≥10 points could help diagnose patients requiring oxygen administration within seven days with a sensitivity/specificity of 59%/97% and a positive/negative likelihood ratio of 18/0.4. With the prior probability of 3.77%, the positive/negative predictive value at a cutoff of 10 points was calculated to be 41.9%/1.63%. Given these results, we propose a risk category classification with a risk score of <5 points as a low-risk group, 5-10 points as a moderate-risk group, and >10 points as a high-risk group. In this study, the proportion of patients requiring oxygen administration within seven days was 0.0% (0/367) in the low-risk group, 4.8% (9/186) in the moderate-risk group, and 42% (13/31) in the high-risk group (Table 4).

**Table 4:** Clinical risk score for predicting oxygen administration required within seven days

Evaluation Items of Clinical Risk Score		No. of Risk Points for Oxygen Administration Required
Body temperature	<37.5℃	0
	37.5℃-38.4℃	4
	≥38.5℃	7
Age	<70 yr	0
	≥70 yr	5
Respiratory rate	<20/min	0
	≥20/min	2
SpO_2_	≥96%	0
	<96%	1

Based on this finding, we suggest that in clinical practice, the low-risk group need not be hospitalized, the moderate-risk group should be considered for hospitalization, and the high-risk group should be required to be hospitalized.

In this study, we established that body temperature, advanced age, increased respiratory rate, decreased SpO_2_, and the presence of pneumonia on CT scans are significant predictors of the need for oxygen within seven days among COVID-19 patients. Our findings underscore the efficacy of a risk score based solely on vital signs, which simplifies the assessment process and effectively predicts the necessity for oxygen therapy with high accuracy (AUC of 0.937, 95% CI: 0.900-0.974). Our research demonstrates that vital signs alone can provide a reliable basis for predicting oxygen requirements in COVID-19 patients, without the need for extensive diagnostics like CT scans. The risk score, which incorporates temperature, age, respiratory rate, and SpO_2_, can be used to make informed triage decisions, facilitating timely and appropriate medical interventions. Given the challenges associated with accessing advanced imaging tools in many healthcare settings, our risk score offers a practical alternative for resource-limited environments. It allows healthcare providers to efficiently identify patients at risk of deterioration and prioritize hospital admissions, potentially reducing the burden on healthcare facilities and improving patient outcomes. Further studies are needed to validate this risk score in broader and more diverse populations to confirm its applicability and reliability across different clinical settings. Additionally, expanding the sample size could enhance the robustness of the findings and help refine the risk assessment criteria to better serve global healthcare needs.

Limitations

When examining the variables associated with oxygen administration required within seven days, the sample size was insufficient. After propensity score matching, there were 38 eligible patients in the group not requiring oxygen administration within seven days and 19 in the group requiring oxygen administration within seven days. This may have biased the target population’s characteristics or resulted in a lack of power to detect a significant association when it was truly relevant. Further investigation with a larger sample size is warranted. This study has another limitation that the patient population used to create the scoring system in this study is the same as the one used to create the ROC curve. When examining the risk score for oxygen administration within seven days, 3.77% (22/584) of patients in the study required oxygen administration within seven days. However, the proportion of patients requiring oxygen administration within seven days in the general population is likely lower, as the patients in this study were referred to our hospital with inpatient facilities. Therefore, the positive predictive value for a cutoff value of 5 or 10 points in the general population may be lower than that calculated in this study, while the negative predictive value may be higher. The validity of this risk score for predicting oxygen administration required in primary care clinics and hospitals remains to be demonstrated.

## Conclusions

This study validated that body temperature, advanced age, increased respiratory rate, decreased SpO_2_, and pneumonia on CT scans significantly predict the need for oxygen within seven days in COVID-19 patients. Our risk score, based only on vital signs, accurately forecasts oxygen requirements (AUC 0.937, 95% CI: 0.900-0.974), offering a practical tool for healthcare providers, especially in settings where access to advanced diagnostics is limited. Further validation in a larger, diverse population is necessary to confirm its broader applicability.

## Figures and Tables

**Figure 4:**
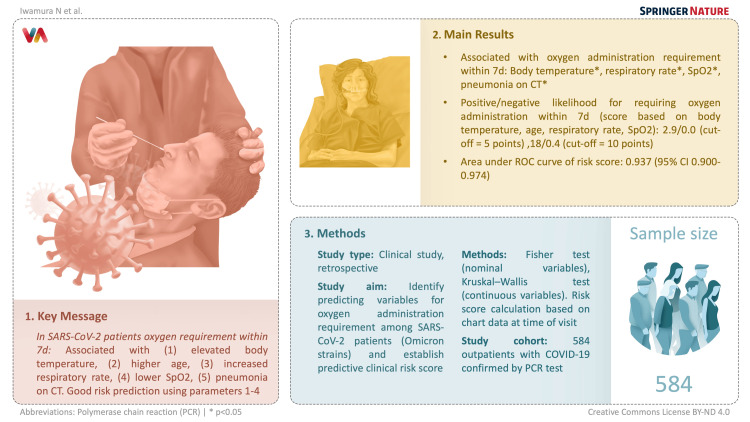
Visual abstract In SARS-CoV-2 patients, oxygen requirement within seven days is associated with (1) elevated body temperature, (2) higher age, (3) increased respiratory rate, (4) lower SpO2, (5) pneumonia on CT. Good risk prediction using parameters. Adapted with permission from Springer Nature Research Solutions and licensed under a Creative Commons License BY-ND 4.0.
